# Physiological Responses of Continuous and Intermittent Swimming at Critical Speed and Maximum Lactate Steady State in Children and Adolescent Swimmers

**DOI:** 10.3390/sports7010025

**Published:** 2019-01-18

**Authors:** Ioannis S. Nikitakis, Giorgos P. Paradisis, Gregory C. Bogdanis, Argyris G. Toubekis

**Affiliations:** 1 Division of Aquatic Sports, School of Physical Education and Sport Science, National and Kapodistrian University of Athens, Dafne, 17237 Athens, Greece; inikitak@phed.uoa.gr; 2 Sports Performance Laboratory, School of Physical Education and Sport Science, National and Kapodistrian University of Athens, Dafne, 17237 Athens, Greece; gparadi@phed.uoa.gr (G.P.P.); gbogdanis@phed.uoa.gr (G.C.B.)

**Keywords:** continuous swimming, intermittent swimming, aerobic endurance, exercise intensity domains

## Abstract

Background: The purpose of this study was to compare physiological responses during continuous and intermittent swimming at intensity corresponding to critical speed (CS: slope of the distance vs. time relationship using 200 and 400-m tests) with maximal lactate steady state (MLSS) in children and adolescents. Methods: CS and the speed corresponding to MLSS (sMLSS) were calculated in ten male children (11.5 ± 0.4 years) and ten adolescents (15.8 ± 0.7 years). Blood lactate concentration (BL), oxygen uptake ($\overset{\cdot }{V}$O_2_), and heart rate (HR) at sMLSS were compared to intermittent (10 × 200-m) and continuous swimming corresponding to CS. Results: CS was similar to sMLSS in children (1.092 ± 0.071 vs. 1.083 ± 0.065 m·s^−1^; *p* = 0.12) and adolescents (1.315 ± 0.068 vs. 1.297 ± 0.056 m·s^−1^; *p* = 0.12). However, not all swimmers were able to complete 30 min at CS and BL was higher at the end of continuous swimming at CS compared to sMLSS (children: CS: 4.0 ± 1.8, sMLSS: 3.4 ± 1.5; adolescents: CS: 4.5 ± 2.3, sMLSS: 3.1 ± 0.8 mmol·L^−1^; *p* < 0.05). $\overset{\cdot }{V}$O_2_ and HR in continuous swimming at CS were not different compared to sMLSS (*p* > 0.05). BL, $\overset{\cdot }{V}$O_2_ and HR in 10 × 200-m were similar to sMLSS and no different between groups. Conclusion: Intermittent swimming at CS presents physiological responses similar to sMLSS. Metabolic responses of continuous swimming at CS may not correspond to MLSS in some children and adolescent swimmers.

## 1. Introduction

Assessment of aerobic endurance and adjustment of training pace in swimming requires testing for the determination of the speed corresponding to well-known indices such as lactate threshold, maximum lactate steady state (MLSS), and $\overset{\cdot }{V}$O_2_max [1,2]. An additional, easy-to-calculate aerobic endurance index is critical speed (CS), which is considered to be the speed swimmers can maintain without exhaustion [3,4] and is a suitable intensity to improve aerobic capacity [5,6]. However, previous studies have shown that CS does not correspond to a tolerable intensity and cannot be sustained for a long time for all swimmers (e.g., 30–40 min; [7,8]). A long-duration training set (e.g., 30–40 min) with appropriate sustained intensity is suggested for the improvement of aerobic endurance [9]. Then, CS intensity should be sustained for a long duration to achieve improvements in aerobic capacity. Moreover, this intensity can also be used during intermittent exercise [10], but the physiological responses and the intensity domain during continuous and intermittent training relative to CS have not been fully elucidated. In addition, these responses may be age-dependent, since children and adolescent swimmers have been shown to present different physiological responses during intermittent and continuous exercise [11,12,13].

The variability in metabolic responses observed when swimming at intensity relative to CS is also highly depended on the distances selected and the mathematical model used for its calculation [1,8]. It is suggested that the duration of selected distances for CS calculation should be between 3 to 10–15 min, to comply with the theoretical definition of the critical power model (exercise without fatigue or exhaustion) [1]. However, previous studies in swimming have used distances of 200 and 400-m or shorter to calculate CS [5,14,15] despite that using short duration distances may overestimate CS [16]. Whatever the case, CS calculated by 200 and 400-m tests is an easier and less time-consuming approach to apply compared to testing several long-duration distances (i.e., swimming 800 or 1500-m).

To characterize the exercise intensity domain of CS, researchers have evaluated blood lactate concentration (BL) and oxygen uptake ($\overset{\cdot }{V}$O_2_) responses during continuous and intermittent swimming at various intensities above and below the speed corresponding to CS [5,7,12,14,15,16,17]. Swimming at “heavy” intensity domain presents increased but steady BL and $\overset{\cdot }{V}$O_2_ responses, while swimming at “very heavy” exercise intensity domain causes a progressive increase in BL and $\overset{\cdot }{V}$O_2_ slow component without attainment of maximum oxygen uptake at exhaustion. Exercise intensity that leads to maximum oxygen uptake attainment and high BL values is in the “severe” intensity domain [2]. In adult swimmers, it seems that BL and $\overset{\cdot }{V}$O_2_ remain steady during continuous swimming below CS, while during swimming above CS there is a progressive increase in BL and $\overset{\cdot }{V}$O_2_, reaching $\overset{\cdot }{V}$O_2_ peak at the end of exercise [7]. These physiological responses characterize the “heavy” and “severe” exercise intensity, respectively. Swimming at a speed corresponding to CS causes a rise in BL and attainment of $\overset{\cdot }{V}$O_2_ peak at exhaustion [7]. Due to the difficulty of continuously collecting expired air during swimming [1], only two studies have provided data of $\overset{\cdot }{V}$O_2_ responses during recovery in adults [7] and adolescents [8], but no study has examined $\overset{\cdot }{V}$O_2_ during intermittent swimming at speed corresponding to CS. In previous studies, adolescents showed steady $\overset{\cdot }{V}$O_2_ responses without $\overset{\cdot }{V}$O_2_ peak attainment at the end of continuous swimming corresponding to CS (~82% $\overset{\cdot }{V}$O_2_ peak; [8]) while adults attained $\overset{\cdot }{V}$O_2_ peak at exhaustion [7]. No study has provided data of $\overset{\cdot }{V}$O_2_ responses in children during continuous and intermittent swimming at speed corresponding to CS. $\overset{\cdot }{V}$O_2_, combined with BL values provide the information to characterize the exercise intensity domain helping coaches to design training sets at an appropriate intensity. Besides $\overset{\cdot }{V}$O_2_, BL is easier to measure in all age-groups during intermittent swimming. Swimming at CS intensity children presented steady BL [18] while adolescents and adults presented an increase of 0.8 and 1.4 mmol∙L^−1^ in BL respectively, from the 10th min to the end of continuous swimming exhausting them in less than 30 min [7,8]. Similarly, a steady BL of 4–5 mmol·L^−1^ was observed in children during intermittent swimming, [12] while adolescents presented a rise in BL after 1200-m in an intermittent set of 400-m repetitions at intensity corresponding to CS [12,14,17]. A likely attenuated fatigue profile, metabolic response and differences in energy contribution in children compared to adolescent and adults may partly explain these differences [11,13].

It should be noted that CS in all the above-mentioned studies was calculated with different combinations of distances and mathematical models. Shorter distances used for CS calculation may overestimate its value and alter subsequent metabolic responses when swimming at overestimated CS. Additionally, no study has examined physiological responses at an intensity corresponding to CS calculated by 200 and 400-m tests compared to MLSS. Comparing the physiological responses of CS to MLSS will help to clarify its importance for training purposes. Previous studies have used an intermittent protocol to obtain the MLSS speed and compare the physiological responses with swimming at speed corresponding to CS obtained by 200 and 400-m tests [5], but such a comparison has limited validity. Therefore, the purpose of this study was to evaluate physiological responses of continuous and intermittent swimming at speed corresponding to CS obtained by 200 and 400-m tests and MLSS in children and adolescent swimmers.

## 2. Methods

### 2.1. Participants

Ten boys (age: 11.5 ± 0.4 years, body mass: 40.0 ± 5.4 kg, height: 149.2 ± 1.5 cm) and ten adolescents (age: 15.8 ± 0.7 years, body mass: 65.9 ± 7.5 kg, height: 177.1 ± 8.4 cm) participated in the study. The swimmers’ best performance in 200-m front crawl represented 90.5 ± 5.3% of the best national performance in the children age-group (160.2 ± 7.4 s, 261 ± 38 FINA points), and 88.7 ± 3.7% of the national record in their respective age-group of adolescents (127.3 ± 5.4 s, 519 ± 64 FINA points). Both children and adolescents were trained 5–6 times per week, with duration of approximately 2 h per session and covering about 3500–4000 m and 5500–6500 m per session, respectively. All tests were carried out during the specific preparation mesocycle. The anthropometric characteristics (body mass, height and sitting height) were measured on the first day. These characteristics were used for the calculation of the age corresponding to peak height velocity (PHV), based on the equation proposed by Mirwald et al. [19]. Children were 2.3 ± 0.3 years before PHV and adolescents 2.1 ± 0.7 years after the age of PHV. All tests were conducted within one month and with at least one-day difference between tests for each swimmer, in a 25-m indoors swimming pool, with constant water and environmental temperature (25 °C, 27–28 °C respectively). Participants were asked to record their diet the day before the first testing session and repeat a diet of similar nutritional content the day before all subsequent testing sessions. The swimmers agreed to participate, and parents or guardians signed a written informed consent before the commencement of the study that had received approval from the institutional ethical committee (1031/6/12/2017).

### 2.2. Calculation of CS

Before each test, a standardized warm up was applied (400-m front crawl, 200-m front crawl drills, 4 × 50-m front crawl at pace of personal best 400-m). After the warm up and before the test began, 8 min of passive rest was allowed. On the first visit, two all-out efforts of 200-m and 400-m with a push off start within the water were carried out to determine CS (the slope of the regression line between distance and time [3]) and $\overset{\cdot }{V}$O_2_ peak. A 30 min recovery period was allowed between tests (5 min of active and 25 min of passive rest). Time to complete each distance was recorded by two independent timekeepers using a digital stopwatch (FINIS 3X300, Finis Inc., Livermore, CA, USA). Before and after the 400-m all-out effort BL was determined in a blood sample taken from the fingertip (Lactate Scout^+^, Leipzig, Germany). Immediately after the 200 and 400 m tests, a breathing mask was fitted on the face and expired air was analyzed using a portable gas analyzer (VO_2000_ Med Graphics, Saint Paul, MN, USA) [20]. The highest value measured after 200 or 400-m, was considered as $\overset{\cdot }{V}$O_2_ peak for each swimmer. Speed at $\overset{\cdot }{V}$O_2_ peak (s$\overset{\cdot }{V}$O_2_ peak) was defined as the average speed at the test in which the highest $\overset{\cdot }{V}$O_2_ peak was attained. Heart rate (HR) was measured during tests (Polar V800, Polar Electro Oy, Kempele, Finland), and peak HR (HRpeak) was defined as the highest HR observed.

### 2.3. Continuous Swimming Tests—Determination of MLSS

For the determination of the MLSS participants performed, in separate days, two to four continuous 30 min swimming efforts at a constant submaximal speed. On the first day, the pace was set at the speed corresponding to the individual CS. When a steady BL was observed in the CS test, the speed increased by 2% at the next visit. When no steady BL was observed in the CS test or the participant failed to complete 30 min of continuous swimming, the speed at the next test decreased by 2%. A blood sample was taken at the start, at the completion of the 10th min and at the end of the test for the determination of BL. Expired air was also collected at the same time points for the determination of $\overset{\cdot }{V}$O_2_ as it is described in the previous paragraph. Each test was terminated after volitional fatigue, or when swimmers were not able to maintain the required speed for two consecutive 25-m laps despite strong verbal encouragement (i.e., slower by 1 s in a 50 m lap). The speed at MLSS (sMLSS) was determined, using the criteria set by Beneke et al. [21] (i.e., the speed where the lactate concentration increased no more or equal to 1.0 mmol∙L^−1^ between the 10th and 30th min of exercise). The average BL between the 10th and 30th min of the MLSS test was defined as BL at MLSS. HR was recorded continuously during all tests and the average HR during the last 30 s before the end of each test was defined as end-HR. A 45-s rest interval was allowed at the 10th min to measure BL and $\overset{\cdot }{V}$O_2_. A pacing device emitting sound signals was attached under the swimmers’ cap to help them maintain the pre-defined speed in each trial (Finis tempo trainer pro, Finis Inc., Livermore, CA, USA). In addition, an assistant was walking alongside the pool at the pre-defined speed of the test, and participants were instructed to keep their head at the level of assistant’s feet.

### 2.4. Intermittent Swimming at CS

At the last visit to the swimming pool, volunteers were required to swim a set of ten 200-m repetitions (10 × 200-m) at a speed corresponding to CS, with a 35–45 s rest interval after each repetition (mean rest duration: 43.17 ± 6.53 s). BL was measured before the start (pre) and after the 2nd, 4th, 6th, 8th and 10th repetition. $\overset{\cdot }{V}$O_2_ was measured before the start of the set (pre) and after each 200-m repetition. HR was recorded continuously during the test, and the average value of the last 30 s of each 200-m repetition was used for the data analysis. To maintain the pre-defined speed, the same pacing device as in continuous swimming was used.

### 2.5. Statistical Analysis

Statistica v.10 software (Stat-Soft Inc, Tulsa, OK, USA) was used for data analysis. Sphericity was verified using Mauchly’s test. When the assumption of sphericity was not met, the significance of F-ratios was adjusted according to the Greenhouse–Geisser procedure. A Student’s *t*-test for independent samples was used to establish differences at $\overset{\cdot }{V}$O_2_ peak, s$\overset{\cdot }{V}$O_2_ peak and time to exhaustion at CS between groups. A 2-way analysis of variance (ANOVA) for repeated measures was used to examine differences between CS and sMLSS (two groups × two tests) and a 3-way ANOVA for physiological responses (BL, $\overset{\cdot }{V}$O_2_ and HR; two groups × two tests × two times of measurement). When significant main effects were found, a Tukey’s honest significant difference (HSD) post-hoc test was used to identify differences between means. Pearson’s correlation coefficient was used to assess association between variables. The effect size (ES) for paired comparisons was calculated with Cohen’s d using the pooled standard deviation as denominator. The ES was considered small if the absolute value of Cohen’s d was less than 0.20, medium if it was between 0.20 and 0.50 and large if it was greater than 0.50 [22]. 95% confidence limits (95% CL) were also calculated. Significance was set at *p* ≤ 0.05. Data are presented as mean ± SD.

## 3. Results

### 3.1. Performance, Peak Heart Rate and Peak Oxygen Uptake in 200 and 400-m Tests

Performance time in 200 and 400-m all-out efforts used for CS calculation was longer in children compared to adolescents (children: 171.3 ± 9.7, 355.1 ± 21.0 s, adolescents: 136.5 ± 6.5, 288.9 ± 13.9 s, *p* < 0.05). HRpeak was higher in children compared to adolescent (193 ± 10 vs. 185 ± 8 b∙min^−1^, ES = 0.96, large t = 2.13, *p* < 0.05). $\overset{\cdot }{V}$O_2_ peak and s$\overset{\cdot }{V}$O_2_ peak were higher in adolescents compared to children (59.4 ± 3.8 vs. 52.3 ± 6.3 mL·kg^−1^·min^−1^, ES = 1.4, large, t = −3.01, *p* < 0.05 and 1.441 ± 0.053 vs. 1.153 ± 0.083 m·s^−1^, ES = 4.2, large, t = −9.26, *p* < 0.05).

### 3.2. Speed and Physiological Responses during Continuous Swimming at CS and sMLSS

CS and sMLSS were higher in adolescents compared to children (*p* < 0.05). However, no difference was observed between CS and sMLSS in children and adolescents (Table 1, F_1,18_ = 2.672, *p* > 0.05). CS and sMLSS corresponded to 200-m swimming time of 183.8 ± 12.0 and 185.3 ± 11.1 s in children and to 152.5 ± 8.2 and 154.4 ± 6.8 s in adolescents, respectively. CS was correlated with sMLSS (r = 0.96, *p* < 0.05) and s$\overset{\cdot }{V}$O_2_ peak (r = 0.93, *p* < 0.05).

**Figure 1:**
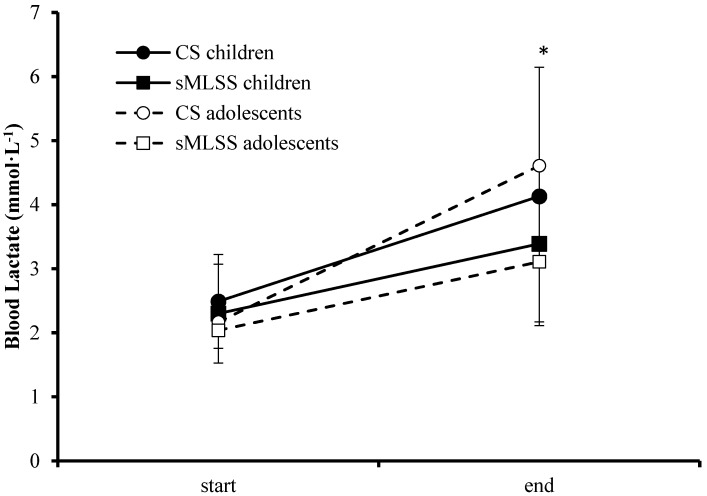
Blood lactate concentration at the start and after the end of continuous swimming at critical speed (CS) and speed corresponding to maximum lactate steady state (sMLSS) in children (black-filled dot and square) and adolescents (white filled dot and square). *: *p* < 0.05, CS compared to sMLSS in both groups.

**Figure 2:**
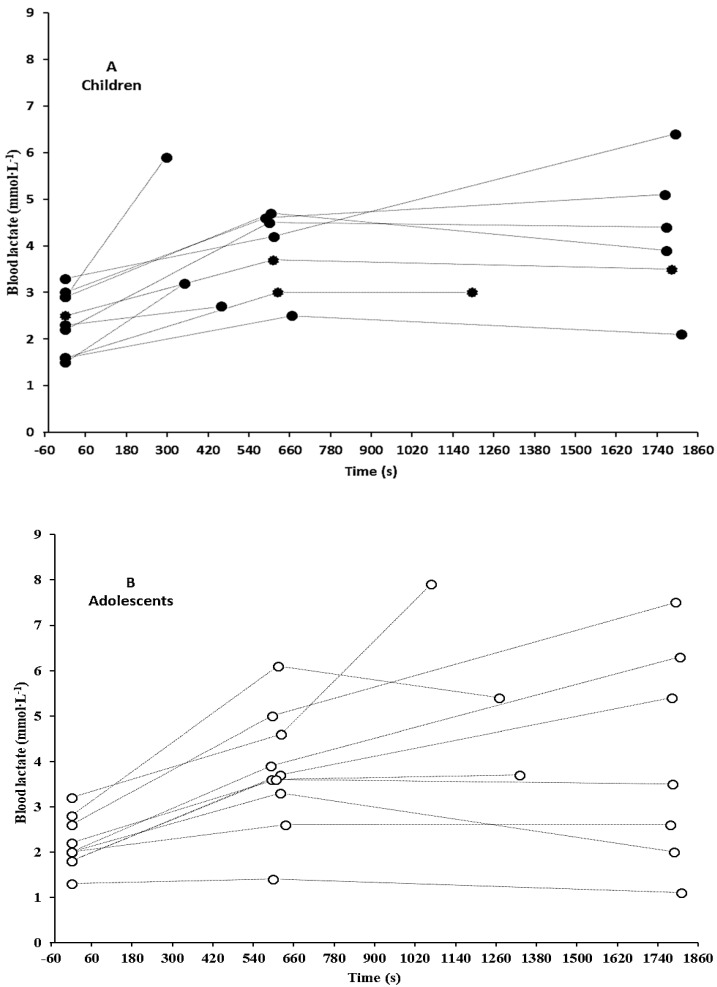
Blood Lactate concentration at start, min 10 and at the end of continuous effort at speed corresponding to critical speed in children ((**A**), filled bullets) and adolescents ((**B**), open bullets).

Mean time to exhaustion during continuous swimming at intensity corresponding to CS was not different between groups (children: 21.2 ± 12.1, 95% CL: 13.7–28.7 min; adolescents: 25.7 ± 7.4, 95% CL: 21.0–30.3 min, t = −0.998, *p* > 0.05). Specifically, six out of ten children and seven out of ten adolescents were able to complete 30 min of swimming at speed corresponding to CS. Five out of six children and four out of seven adolescents who were able to complete 30 min of swimming at CS presented steady BL between the 10th and 30th min. Three children were not able to complete 10 min of swimming at CS. A significant correlation was observed between distance covered at CS and CS as percentage of the 400-m speed (r = −0.71, *p* < 0.05). Analysis of variance showed significant interaction of test × time for BL (F_1,18_ = 563, *p* < 0.05), with continuous swimming at CS resulting in higher BL than sMLSS in both children and adolescents (Figure 1). Figure 2 illustrates individual BL values for all participants at the start, at minute 10 and the end of continuous swimming in speed corresponding to CS.

$\overset{\cdot }{V}$O_2_ was not different in adolescents compared to children at the end of continuous swimming at CS (F_1,18_ = 2.464, *p* > 0.05; Table 1) and not different during continuous swimming at CS compared to sMLSS (F_1,18_ = 1.126, *p* > 0.05; Table 1). HR was similar during continuous swimming at CS compared to sMLSS (F_1,18_ = 3.238, *p* > 0.05) but increased in children compared to adolescents (F_1,18_ = 5.631, *p* < 0.05; Table 1).

### 3.3. Physiological Responses during Intermittent Swimming at CS

**Figure 3:**
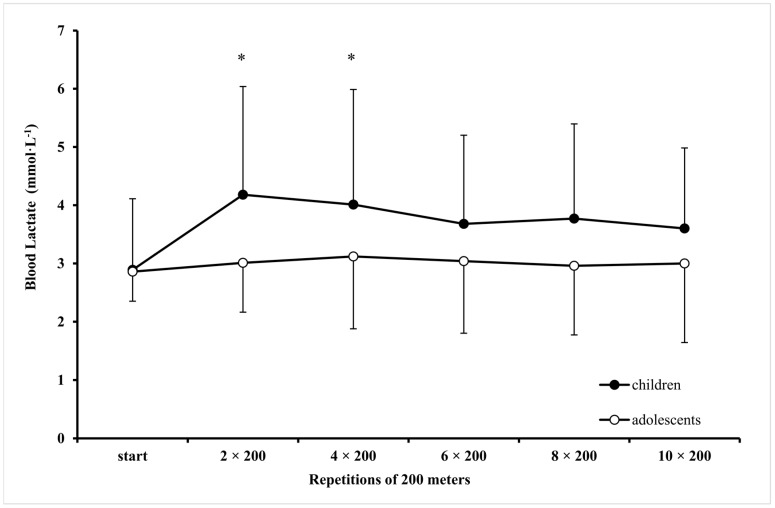
Blood lactate concentration during intermittent swimming (10 × 200-m) at speed corresponding to critical speed in children (filled black bullet) and adolescents (open bullet). *: *p* < 0.05 compared to start in children group only.

**Figure 4:**
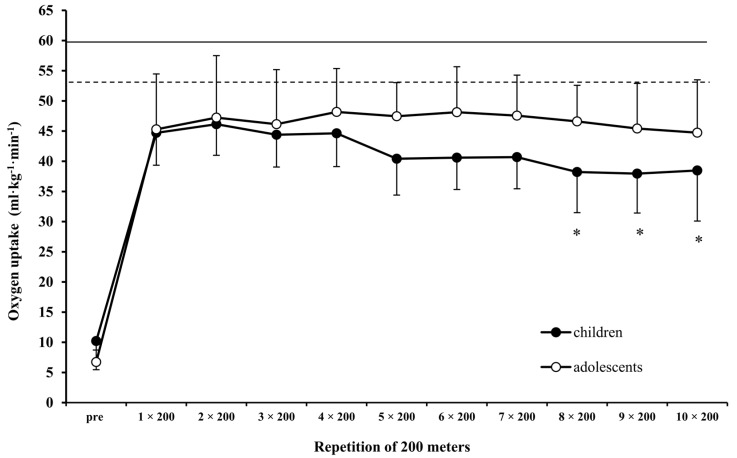
Oxygen uptake during intermittent swimming (10 × 200-m) at speed corresponding to critical speed in children (black-filled bullet) and adolescents (open bullets). *: *p* < 0.05 compared to first and second repetition in children group only. The horizontal dotted and continuous lines show the $\overset{\cdot }{V}$O_2_ peak values of children and adolescents, respectively.

Both children and adolescents maintained a speed equal to CS during the ten 200-m repetitions in the intermittent swimming test (mean speed at 10 × 200-m; children: 1.093 ± 0.067, adolescents: 1.323 ± 0.063 m·s^−1^; CS vs. mean speed at 10 × 200-m F_1,18_ = 1.426, *p* > 0.05). BL remained unchanged during the 10 × 200-m repetitions and was not different between groups (*p* > 0.05, Figure 3). Mean BL during the 10 × 200-m intermittent swimming was similar to mean BL measured at sMLSS for both children and adolescents (10 × 200-m, children: 3.8 ± 1.6, adolescents: 3.0 ± 1.1; sMLSS, children: 3.5 ± 1.5, adolescents: 3.0 ± 0.7; F_1,18_ = 0.247, *p* > 0.05). $\overset{\cdot }{V}$O_2_ was not different between groups (F_1,18_ = 2.818, *p* > 0.05). However, $\overset{\cdot }{V}$O_2_ remained unchanged during 10 × 200-m in adolescents but decreased after the eighth compared with the first bout in children (F_10,180_ = 4.891, *p* < 0.05, Figure 4). HR was similar between children and adolescents (183 ± 2 vs. 173 ± 3 beats·min^−1^, F_1,18_ = 3.973, *p* = 0.06) and remained unchanged over the 10 × 200-m repetitions.

## 4. Discussion

The purpose of this study was to evaluate physiological responses during continuous and intermittent swimming at speeds corresponding to CS calculated by 200 and 400-m tests and MLSS in children and adolescent swimmers. The main findings showed that: (i) CS was similar to sMLSS in both children and adolescents, (ii) intermittent but not continuous swimming at critical speed was sustainable for children and adolescent swimmers, (iii) the physiological responses during continuous swimming at CS indicate that this intensity corresponds to MLSS for some children and adolescent swimmers but it may be above or below MLSS by 6% and 4% respectively for others.

CS was not different compared to sMLSS in both children and adolescents. Previous studies calculated CS by the time to exhaustion (tests duration of 2 to 7 min) or by 200 and 400 m tests reported a higher CS compared to sMLSS in adult swimmers of similar performance level compared to adolescent swimmers in the present study [23,24]. In contrast, studies conducted with children and adolescent swimmers agree with our findings, showing CS similar to sMLSS [15,18]. It is likely that the duration of selected distances may be shorter in adults compared to younger swimmers, thus overestimating CS (the slope of distance vs. time). Moreover, the speed difference between 200 and 400-m within each group will also impact CS calculations. In the present study children were slower by 20.3% and 18.3% in 200-m and 400-m tests compared to adolescents. It is also interesting to note that the speed difference in 200 vs. 400-m in children was 3.5% compared to 5.5% in adolescents. A 5.5% difference has been also observed in adult swimmers [23]. The observed differences may be attributed to an attenuated metabolic acidosis and fatigue combined with increased reliance on aerobic metabolism in children compared to adolescents and adults [11,13,25]. Altogether the above-mentioned observations highlight the need for a careful selection of distances and the mathematical model for CS calculation especially when a comparison between age-groups or between CS and MLSS is attempted.

In the present study CS and sMLSS differed by 0.8% and 1.3% in children and adolescent swimmers, respectively. However, a great variability between swimmers in both groups was observed. Some of the swimmers were able to swim 30 min with a steady lactate concentration at intensity 4% faster than CS while others should have decreased speed for about 6% to maintain steady lactate responses from the 10th to the 30th min of continuous swimming. This information may explain why some swimmers were not able to complete 30 min at CS intensity. Another possible reason that explains the inability of some children and adolescents to complete 30 min at CS intensity is that these swimmers presented a CS over 96.5% of the 400-m speed and there was a strong relationship between distance covered at CS intensity and CS as % of the speed at 400-m. Previous studies agree with our findings that continuous swimming at CS can be maintained for 24 to 27 min, albeit with a great variability (standard deviations of 5 to 7 min) [7,8], as was also the case in both children and adolescents groups in the present study. However, it seems that 10–12 years old children can complete 30 min swimming at a speed corresponding to CS, while maintaining a steady blood lactate concentration [18]. These differences may appear because of different combination of distances and the mathematical model used for CS calculation leading to overestimation or underestimation of CS [1]. The combination of distances and the two-parameter linear model that used in the present study may overestimate CS compared to other models and combinations that include longer distances for CV assessment [8]. 

A high relative percentage of CS compared with the maximum aerobic speed (92–94%) and a difference of 5–8% between sMLSS and CS have also been reported in previous studies [23,24]. The CS measured in the present study corresponded to 95% and 97% of the speed at 400-m for adolescent and children swimmers, respectively. This may explain why, despite a small mean difference between CS and sMLSS, this speed was not sustainable for most children and adolescent athletes, as they had to swim at a speed very close to their best 400-m performance. Furthermore, children swimmers participated in the present study presented faster CS and sMLSS compared to previous study [18], then, they had to apply more power to overcome drag force than lower level swimmers at the same intensity relative to maximal aerobic speed [26]. This information may explain why a small difference between speeds of about 1%, but with a range of −6.4% to 4.3% for children and −6.1% to 4.5% for adolescents, leads to different physiological responses when aiming to maintain CS intensity for 30 min compared to sMLSS. Swimmers in previous studies, to continue swimming for 30 min, adjusted their speed to a lower intensity (~96–97% of CS, [14,24]). In the present study, all participants were able to comply with the imposed speed (the CS), but some of them failed to maintain it for 30 min. Thus, our findings suggest that great inter-individual differences may appear between CS and sMLSS (−6% to 4%) that may critically alter sustainability or metabolic responses despite that the range of mean speeds corresponding to various aerobic indices is very narrow in swimming [27]. Moreover, the selection of distances for CS calculation in the present study (200 and 400-m) may overestimate the “ideal” CS calculation and this should be considered as a limitation of the current study.

CS and sMLSS did not differ from each other statistically; however, it is possible that these aerobic indices represent different exercise intensities, due to different individual physiological responses observed during swimming at these intensities. This is confirmed by the higher lactate responses and small to medium increments in end-HR and $\overset{\cdot }{V}$O_2_ observed during continuous swimming at CS compared with sMLSS, in combination with the shorter time to exhaustion observed in continuous swimming at CS compared to the standard time of 30 min at sMLSS. Blood lactate concentration compared to previous studies [7,8] was not as high as expected during the CS intensity but was higher than the concentration measured in the sMLSS speed. Adolescent swimmers in previous studies completed continuous swimming or terminated the test with higher blood lactate concentration of 6 to 7 mmol∙L^−1^ [7,8]. However, our measured values in children’s group were comparable to previous study with children swimmers (e.g., 3 to 4 mmol∙L^−1^, [18]). It is possible that at intensities above MLSS, (i.e., CS in some swimmers in the present study), exercise termination may be induced by protective mechanisms to ensure the maintenance of homeostasis, even though lactate concentration is not very high ~4 mmol·L^−1^ and $\overset{\cdot }{V}$O_2_ peak is not reached [28]. Thus, CS calculated by 200 and 400-m seems to be a hardly tolerable intensity, which some swimmers cannot maintain for a long time (e.g., 30 min [7]). Children and adolescents did not reach $\overset{\cdot }{V}$O_2_ peak during the continuous swimming at CS intensity as it has been reported in a previous study with adult swimmers [7]. This means that CS probably does not represent the boundary between “very heavy” and “severe” exercise intensity domain. Furthermore, CS may represent slightly higher intensity in children compare to adolescents, due to the higher cardiovascular effect (higher heart rate) observed in continuous swimming. Children compared to adolescents and adults present lower stroke volume and they need to increase their HR to maintain cardiac output [29].

In contrast to continuous swimming, children and adolescents were able to maintain CS during the 10 × 200-m interval training, with physiological responses similar to sMLSS. Previous studies have shown that swimming at CS induces an increase in blood lactate concentration after 800 m [12] or 1200 m [14,17] in a series of 5 × 400-m. However, children have shown steady lactate concentration during repetitions of 300 m at CS intensity [12]. Nevertheless, in the present study, lactate concentration showed steady levels in both, children and adolescents during the 10 × 200-m intermittent swimming. To explain this disagreement, we should consider that in the above-mentioned studies, adolescents performed repetitions of 400 m in a 50-m swimming pool, while in the present study children and adolescents performed repetitions of 200 m in a 25-m swimming pool, using the same resting interval (e.g., 30–45 s). The shorter duration of repetitions and the shorter pool length may have helped in attenuating metabolic responses [30]. When swimmers perform short-term efforts (up to 200 m), they can maintain steady metabolic conditions more easily compared with longer distances such as the 400-m repetitions [7,14,31,32]. In addition, $\overset{\cdot }{V}$O_2_ also showed a steady state between repetitions in children and adolescents, without attainment of $\overset{\cdot }{V}$O_2_ peak. Furthermore, in accordance with a previous study, HR was steady between repetitions without differences between children and adolescents [12]. Values obtained in the present study were similar to the values obtained in a study during which adolescents exhibited heart rate within the range of 180–197 beats·min^−1^ during various intermittent sets independent of the distances used (i.e., 5 × 400-m, 10 × 200-m, 20 × 100-m) [14].

## 5. Conclusions

The speed corresponding to CS calculated by the two-parameter linear model and using distances of 200 and 400-m represent an intensity that not all children and adolescent swimmers are able to maintain for a long period during continuous swimming. Considering the time to exhaustion, the metabolic, $\overset{\cdot }{V}$O_2_ and heart rate responses observed in children and adolescent swimmers in the present study, it seems that CS obtained by 200 and 400-m tests represents exercise intensity above the “heavy” domain for some of the swimmers. On the other hand, 30–45 s passive recovery between 10 × 200-m swimming repetitions enables steady lactate concentration, $\overset{\cdot }{V}$O_2_ and HR similar to MLSS. Swimming coaches may use 200-m swimming repetitions with rest interval of 30–45 s at a pace corresponding to CS calculated by 200 and 400-m aiming to improve aerobic endurance of well-trained children and adolescent swimmers.

## Figures and Tables

**Table 1:** Mean ± SD and 95% confidence limits for heart rate (HR) and oxygen uptake ($\overset{\cdot }{V}$O_2_) at the end of continuous swimming at critical speed (CS) and at the speed corresponding to maximum lactate steady state (sMLSS).

Variable	Children			Adolescents		
	CS	sMLSS	ES	CS	sMLSS	ES
Speed (m·s^−1^)	1.092 ± 0.071 ‡ (1.136–1.048)	1.083 ± 0.065 ‡ (1.123–1.042)	0.14 small	1.315 ± 0.068 (1.357–1.237)	1.297 ± 0.056 (1.332–1.263)	0.29 medium
End-HR (b·min^−1^)	188 ± 13 ‡ (180–196)	187 ± 8 ‡ (182–191)	0.18 small	179 ± 9 (174–185)	175 ± 12 (168–182)	0.44 medium
End $\overset{\cdot }{V}$O_2_(mL·kg^−1^·min^−1^)	43.3 ± 5.4 (39.9–46.6)	40.7 ± 7.4 (36.1–45.3)	0.40 medium	46.3 ± 6.6 (42.2–50.4)	44.6 ± 6.9 (40.4–48.9)	0.25 medium

ES: effect size between CS and sMLSS in each group, ‡: *p* < 0.05 compared to adolescents (main effect of group).
